# Quality and safety of municipal drinking water in Addis Ababa City, Ethiopia

**DOI:** 10.1186/s12199-020-00847-8

**Published:** 2020-03-09

**Authors:** Amsalu Mekonnen Wolde, Kemal Jemal, Gebru M. Woldearegay, Kassu Desta Tullu

**Affiliations:** 1 Department of Medical Laboratory Sciences, College of Health Sciences, Addis Ababa University, Addis Ababa, Ethiopia; 2 College of Health Sciences, Salale University, Fiche, Ethiopia; 3 Virginia Commonwealth University Health Systems, Richmond, USA

**Keywords:** Bacteriological quality, Municipal water, Coliform level, Safety

## Abstract

**Background:**

In low resourced countries, water-associated diseases have still impact on public health. Poor quality of water can cause waterborne diseases through bacteria, viruses, protozoa, and parasites that has been responsible for millions of morbidity and mortality. Therefore, this study aimed to assess quality and safety of public municipal drinking water in Addis Ababa City.

**Methods:**

Descriptive epidemiological study design that used quantitative approach was carried out at Addis Ababa City Administration from June 2016 to October 2016. Pre-tested and standardized aseptic sample collection technique was utilized to collect a total of 2976 samples (2951 water samples for bacteriological analysis by Presence-Absence (P-A) culturing method and 25 samples for parasites identification through direct microscopy examination). Descriptive data were summarized and cleaned by the SPSS version 20 software and presented in table and graph.

**Results:**

The study revealed that 10%, 7% and 3% were positive for bacteriological, total coliforms, and fecal coliforms respectively through Presence-Absence Broth test. The bacterial distribution trends from 1st to 13th weeks of wet season were slight increment of total coliforms and slight decrement for fecal coliforms. All tested for parasitological samples from selected reservoirs were free from parasitological species.

**Conclusion:**

This study reflects that there were positive for bacterial, total coliforms, and fecal coliforms during the study period. It needs continuous screening and treating water sources to utmost important for prevention and control waterborne disease.

## Background

Globally, contaminated drinking water with microorganism has been lead to serious morbidity and mortality [1]. It serves as a mechanism to transmit communicable diseases such as diarrhea, cholera, dysentery, typhoid, and guinea worm infection [2]. In resource limited countries, diseases associated with poor quality of water and sanitation are still have economic impacts on human development and health expenses [3].

In 2013, there were nearly 1.8 million deaths mainly with diarrhea and cholera due to inadequate sanitation and hygiene [4]. Worldwide, diarrhea ranks second after respiratory infections and causes incidence of 4,600 million episodes, and 2.2 million deaths every year, which 15% was under-5 years mortality [5].

In Africa, roughly 40% of the population does not have access to improved water supply and sanitation [6]. Study conducted in rural villages of Mohale Basin in Lesotho show that drinking water was polluted by *Escherichia coli* (78% of unprotected water and 60% of protected water sources) and 59% of water sample contain open defecation with poor control of hygiene practice [7].

In Ethiopia studies conducted in Dire Dawa and Jimma revealed 83.34% and 87.5% of water sample were positive for bacterial indicators respectively [8, 9]. Additionally, a study done in North Gondar showed that springs (35.7%), protected wells (28.6%), and water lines (50%) had *Escherichia coli* [10].

Quality and safety of drinking water is challenging due to contaminants from man-made and natural disasters. Many of the diseases in communities are caused by microorganisms carried in drinking water [11]. The contaminated drinking water is not only due to fecal contamination but also growing in piped water distribution systems [12].

Quality and safety of water supply is a pillar for primary prevention and control of pathogenic microorganisms (bacteria, viruses, protozoa, and helminthes). As a result, chlorination is by far applied as disinfecting methods and destroys many microorganisms and determine the indication of safe water supply that free from bacteria and other organisms [13, 14].

Safety and quality of water supply is an important priority to protect human health and well-being. Addis Ababa has high rainfall during the wet season which may expose drinking water for bacteria and parasites. However, there is a limited assessment concerning to the quality and safety status of water from sources in Addis Ababa during wet season. Therefore, this study aimed to assess the bacteriological and parasitological quality and safety status of all sources of Addis Ababa municipal drinking water during rainy season.

## Methods

### Study area

The study was carried out at Addis Ababa City Administration. Addis Ababa is the capital city of Ethiopia and diplomatic capital of Africa. It is located in the heart of the country surrounded by mountains and 2355 m above sea level. The city covers about 527 km^2^, and approximately 4 million populations live in 10 sub-cities and 116 districts [15].

### Study design, period, and inclusion

Descriptive epidemiological study design that utilizes quantitative approach was conducted from June 01, 2016 to October 31, 2016. All public municipal drinking water service sources managed by Addis Ababa Water and Sewerage Authority (AAWSA) were included.

### Sample collection and procedure

Purposive sampling technique was employed to select drinking water from public taps, service reservoirs, springs, and wells. Observational check lists also used to make sure the handling of water sources. The city administration water system was divided based on the endpoints, hydraulic zone, and topography of distribution system of water sources (reservoirs, springs, and wells).

A total of 2976 samples were collected and examined for bacteriology and parasitology this study (2951 samples for bacteriology and 25 samples for parasitology, one sample for bacteriology = 125 ml of water, and one sample for parasitology = 11 l of water). Using aseptic technique, drinking water was collected according to the WHO, UNICEF, and US EPA sampling methods for bacterial and parasitological examination [16–18].

As quenching agent, 0.1 ml of 3% sodium thiosulfate (Na_2_S_2_O_3_) was added to containers (the sample bottles) before the collection of water having residual chlorine (for treated samples) at the laboratory before sample collection to neutralize the chlorine present in the sample [19].

The bacteriological samples were collected between 9:00 a.m. to 11:00 a.m. in sterilized glass bottles in 13 weeks/rounds from public taps (1833 samples from cafes, factories, hotels, households, offices, restaurants, schools, and university), service reservoirs (429 samples), springs (143 samples), and wells (546 samples) during the wet season (June 01, 2016 to September 8, 2016).

For parasitology, the samples were collected in sterilized plastic jerry cans from three main water plants: Akaki, Gefersa, and Lege Dadi between September 12, 2016 and October 31, 2016. Then, both bacteriological and parasitological samples were labeled with unique code, kept in ice pack, and transported to AAWSA water quality control laboratory department.

## Laboratory testing

### Bacteriological investigation technique

The method we used to examine the presence of bacteria species in public municipal drinking water was presence-absence (P-A) Broth test. The P-A Broth is used for detecting coliforms in a simple modification of the multiple-tube procedure in treated water. Simplification was used for one large test portion (100 ml) in five culture bottles to obtain qualitative information to estimate the presence or absence of coliforms. As soon as P-A test is positive, coliform density can be firmed quantitatively in duplicated samples to point out the magnitude of the contamination. Composition ingredients were used for culture medium. This method was selected due to the possibility of examining a larger number of samples per unit at a time and relatively inexpensive [20].

### Parasitological examination technique

The collected water samples (11 l) were first filtered through a 47-mm diameter, 0.450-μm pore size membrane filters by a pressure of vacuum pump (the standard was 10 to 50 l). The sediments collected on the filter membrane were first transferred into 15-ml conical centrifuge tube containing distilled water and centrifuge at 5000 rpm for 10 min. Finally, the sediment was prepared for direct observation of parasite cysts, trophozoites, and helminthes ova/eggs through microscopy examination [21].

### Quality assurance

To maintained quality control, distilled water from the study laboratory was taken to all sample collection sites and carry along with the water sample back to the laboratory. Then, both water samples were analyzed in parallel through monitoring internal and external quality control. Media, reagents, and samples were run with positive and negative controls under supervision of environmentalist and biologists at AAWSA water quality control.

### Statistical description

The data were entered and cleaned by statistical package for social science (SPSS) version 20.0. Basic descriptive summaries were used to describe measures of central tendencies and dispersion of microbial concentrations. The frequencies and percentages were calculated to evaluate bacterial and parasitological qualities and safety statuses of each water service sources. The results were displayed in tabular and graphic forms.

### Ethical consideration

Ethical clearance approval was obtained from Addis Ababa University College of Health Sciences. Official letter was obtained from AAWSA, and water samples were collected and tested with the unique identification code, and all confidentiality of the results was maintained with great care. Therefore, the confidentiality of the results of all samples was kept from the time of sample collection up to the end of result dissemination.

## Results

The municipal drinking water samples of 2951 were examined for bacteriology, and microorganisms were detected in four municipal drinking water sources of Addis Ababa. Almost 90% of samples were free from contaminated, and 281 (10%) were contaminated. Consequently, 204 (7%) and 77 (3%) samples had total coliforms and fecal coliforms respectively. Moreover, 106 (6%) samples of all public taps, 26 (6%) samples of reservoirs, 34 (24%) samples of springs, and 115 (21%) samples of wells were contaminated with bacteria. The overall bacterial results were found the least in public taps and reservoirs, and the highest results were in springs and wells (Table 1).

**Table 1:** The four sources (public tabs, reservoirs, springs, wells) results of public municipal drinking water in Addis Ababa, 2016

Sample source	Total negative	Total positive	Total coliforms	Fecal coliform positives	Total samples
Public taps	1727 (94%)	106 (6%)	78 (4%)	28 (2%)	1833
Reservoirs	403 (94%)	26 (6%)	22 (5%)	4 (1%)	429
Springs	109 (76%)	34 (24%)	21 (15%)	13 (9%)	143
Wells	431 (79%)	115 (21%)	83 (15%)	32 (6%)	546
Total	2670 (90%)	281(10%)	204 (7%)	77 (3%)	2951

**Fig. 1:**
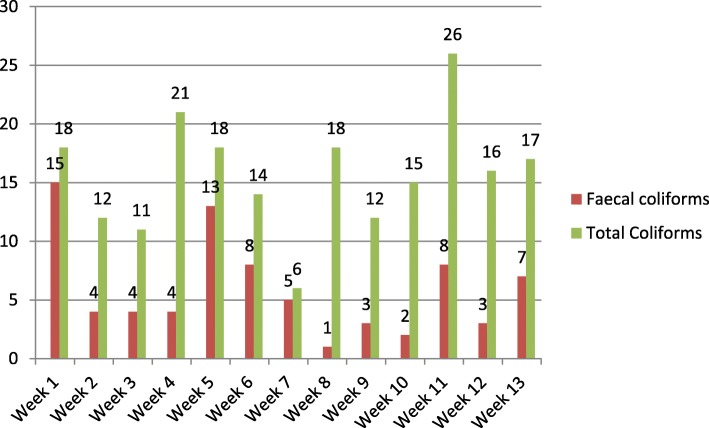
The trends of bacterial distribution during the study period of the wet season in the four sources of Addis Ababa City Administration, 2016

The municipal drinking water was contaminated with both fecal coliforms and total coliforms throughout the sampling rounds of the wet season. The highest fecal coliforms were observed in week 1 and 5 while the highest total coliforms were observed in week 11. There were slight increments of total coliforms and a little decreasing of fecal coliforms from week 1 to week 13 of the wet season. The decrement of fecal coliforms may be due to the rain season decreased from August to September. On the other hand, contamination indicators were varied in weeks of the wet season. The nature and likelihood of pollution can vary seasonally with rainfall and local conditions (Fig. 1).

From Akaki, Gefersa, and Lege Dadi water plants, 25 samples were collected. The parasitological examinations through microscopy examination tests were negative. Thus, the collected samples from reservoirs were free from parasite species.

### Observation

Quality and safety of a water supply was depended upon proper construction and protection. A favorable bacteriological analysis alone should not be accepted as conclusive evidence of the quality and safety of a water supply**.** Therefore, all sites of water supply sources were observed on-site for defects.

It was observed that most service reservoirs were well-handled, but insignificant number of contaminations was detected. On the other hand, most springs were highly exposed to heavy rain, flood, and microorganism contamination. It was also observed that the majority private taps were tied with ropes, plastic tubes, and pieces of clothes which can harbor microorganisms and help to multiply. In some areas of the city, the taps were old and exposed to breakages.

## Discussion

The microbiological examination of drinking water emphasizes on assessment of hygienic and quality of water supply [22, 23]. This requires the isolation and enumeration of organisms that indicate the presence of fecal contamination. Yet, the quality and safety of drinking water was not determined by bacteriological parameters, but also physicochemical content of ion in the water maters for human health [24–26]. In certain circumstances, the same indicator organisms may also be used to assess the efficiency of drinking water treatment plants, which is an important element of quality control [27]. The guideline of WHO places standard of quality and safety drinking water for total coliform bacteria must not be detectable in any 100-ml sample. However, in case of large supplies, where sufficient samples are examined, must not be presented in 95% of samples taken throughout any 12-month period [2].

In this study, we found that about 10% of all bacteriological samples were positive for total coliforms and fecal coliforms. This finding was lower than the studies finding in Jimma and Dire Dawa Ethiopia [8, 9]. This difference may be AAWSA controls the safety and quality of water through evaluation of water sources and manage contamination of water supply.

In our study, 7% of the municipal drinking water samples were contaminated with total coliforms during the study period. This study was in line with the study done in Saudi Arabia which found 12% of drinking water was positive for total coliform [28]. On the other hand, our study was lower than the study in Pakistan [29]. The difference may be the geographical area and the instrument they used to examined.

This study also indicates that 3% of the municipal drinking water samples were contaminated with fecal coliforms. Presence of fecal coliform bacteria signifies fecal contamination of the water supply has occurred. This finding was lower than the studies done in India, Pakistan, and Sierra Leone which showed 78.1%, 70%, and 61% of drinking water were contaminated with *Escherichia coli* and fecal coliforms bacteria respectively [29–31].

In sub-Saharan countries particularly in Ethiopia, drinking water is obtained from different sources, such as taps, reservoirs, springs, and wells. In our results, the municipal drinking water sources of study area were contaminated with microorganisms during the study period. Accordingly, 6% of drinking water collected from public taps (4% total coliforms and 2% fecal coliforms) was positive for bacterial groups. Again, 6% of drinking water collected from service reservoirs (5% total coliforms and 1% fecal coliforms) was positive for bacterial groups. Other similar studies had showed that most collected samples from reservoirs had both total coliforms and fecal coliforms positives [32, 33].

Additionally, 24% of samples from springs (14% total coliforms and 9% fecal coliforms) and 21% samples from wells (15% total coliforms and 6% fecal coliforms) were positive for bacterial species. There is a similar study finding in Saudi Arabia [28]. This finding was lower than the study done in Ethiopia [34]. This variation may occur due to different source of water and place where the study conducted.

Seasonal change can be contaminating the quality and safety of drinking water. Our study undertake data collection during Ethiopian wet season. The maximum total coliform and fecal coliforms of positive samples were seen in week 11 and week 1 respectively. There was a slight variation of total coliforms and fecal coliforms during wet season. Study showed that flash flooding and heavy rainfall were polluted drinking water as a result of *Escherichia coli*, *Shigella*, *Salmonella*, and *Staphylococcus aureus* [35]. A thorough study was done on the basis of prevailing seasons were found highest report of total coliforms in the water sample during wet season [36]. The impact of climate change on water sources was expected to infect water through increased rainfall frequency, intensity, and prolonged rainfall and may present higher *Cryptosporidium* and *Giardia* [37].

Finally, we found that all samples collected for parasitological species examination was negative. This study different from the study conducted in Saudi Arabia that revealed *Giardia cysts* (25%) and *C. parvi oocysts* (16.6%) was detected in water samples both microscopy and ELISA methods [28]. This difference may be due to the water sources differences. In addition, in the study done in Iran, 40% of drinking water samples was infected by Protozoa (28.7%) and *Entamboeba histolostica* (6.3%) [38]. Even though our study was similar methodology with Iran findings, the differences were sample sources.

The limitation of this study was P-A Broth and only depends on wet season. P-A Broth test is simple, contains large sample size, and may not pick enough bacteriological patterns in the drinking water. Even though, the simplicity of P-A method we use suspends 91.53 g in 1000 ml distilled water to prepare a triple strength medium. Second, the study duration was in wet season that may not represent the other season.

## Conclusion

The result from laboratory and observation study indicates that there were low magnitude positive findings of total coliform and fecal coliforms organisms in Addis Ababa water supply. This contamination of water sources was may be due to insufficient chlorination and poor handling of public taps that may affect the quality and safety of drinking water. Therefore, utilization of energy and resources was aimed to protect the public health from both bacteriological and parasitological contaminants that harm the public health and economy. Periodic assessment on the quality and safety of drinking water deserves public health well-being and control measures.

## Abbreviations

AAWSA: Addis Ababa City Administration Water and Sewerage Authority
AAU: Addis Ababa University
UNICEF: United Nations Children’s Fund
USEPA: United States Environmental Protection Agency
WHO: World Health Organization
